# JS-K induces autophagy-dependent ferroptosis in bladder cancer: a multimodal mechanistic and translational study

**DOI:** 10.1093/pcmedi/pbag012

**Published:** 2026-04-25

**Authors:** Zhuo Li, Haitao Zhong, Daniel Baptista-Hon, Jingping Qiu, Zhongwei Zhang, Xinrui Zhang, Yuwan Zhao, Lugang Zhu, Sheng Gao, Bin Li, Olivia Monteiro, Jianjun Liu, Yumin Zhuo

**Affiliations:** Department of Urology, The First Affiliated Hospital of Jinan University, Guangzhou 510630, China; Laboratory of Urology, Affiliated Hospital of Guangdong Medical University, Zhanjiang 524001, China; Department of Urology, Sun Yat-sen memorial Hospital, Sun Yat-sen University, Guangzhou 510120, China; Faculty of Medicine, Macau University of Science and Technology, Macau 999078, China; School of Medicine, University of Dundee, Dundee DD1 4HN, United Kingdom; Macau Institute for Artificial Intelligence in Medicine, Macau University of Science and Technology, Macau 999078, China; Laboratory of Urology, Affiliated Hospital of Guangdong Medical University, Zhanjiang 524001, China; Laboratory of Urology, Affiliated Hospital of Guangdong Medical University, Zhanjiang 524001, China; Laboratory of Urology, Affiliated Hospital of Guangdong Medical University, Zhanjiang 524001, China; Laboratory of Urology, Affiliated Hospital of Guangdong Medical University, Zhanjiang 524001, China; Laboratory of Urology, Affiliated Hospital of Guangdong Medical University, Zhanjiang 524001, China; Laboratory of Urology, Affiliated Hospital of Guangdong Medical University, Zhanjiang 524001, China; Laboratory of Urology, Affiliated Hospital of Guangdong Medical University, Zhanjiang 524001, China; Faculty of Medicine, Macau University of Science and Technology, Macau 999078, China; School of Medicine, University of Dundee, Dundee DD1 4HN, United Kingdom; Macau Institute for Artificial Intelligence in Medicine, Macau University of Science and Technology, Macau 999078, China; Laboratory of Urology, Affiliated Hospital of Guangdong Medical University, Zhanjiang 524001, China; Department of Urology, The First Affiliated Hospital of Jinan University, Guangzhou 510630, China

**Keywords:** bladder cancer, ferroptosis, autophagy, JS-K, *GPX4*, *CISD1*

## Abstract

**Background:**

Bladder cancer is the most common urological malignancy. Bladder cancer has limited therapeutic options, especially in advanced stages. Ferroptosis, an iron-dependent form of regulated cell death, has emerged as a promising target for cancer therapy. However, the role of autophagy in modulating ferroptosis remains incompletely understood.

**Methods:**

We investigated the anti-tumor effects of JS-K, a nitric oxide-releasing prodrug, in bladder cancer through integrated cell, animal, and patient data studies. *In vitro* experiments with T24 and UM-UC-3 cells were used to explore how JS-K influences cancer cell survival and the interplay between autophagy and ferroptosis. *In vivo*, a BALB/c nude mouse tumor model provided a system to examine tumor response and tissue-level changes. To extend these findings to the clinical setting, we analyzed *LC3B* expression and its associations with ferroptosis-related genes, patient prognosis, and the tumor immune microenvironment.

**Results:**

JS-K induced mitochondrial damage, lipid peroxidation, reactive oxygen species accumulation, and intracellular iron overload in bladder cancer cells in a concentration-dependent manner. These changes were accompanied by downregulation of GPX4 and SLC7A11 and upregulation of FTH1 and TFR1, indicative of ferroptosis. Inhibition or knockdown of the autophagy marker *LC3B* reversed these effects, establishing the role of autophagy in mediating ferroptosis. In xenograft models, JS-K suppressed tumor growth, an effect abrogated by *LC3B* silencing. Integrated transcriptomic and single-cell analyses revealed a strong correlation between *LC3B* and ferroptosis-related genes, with *CISD1* identified as a key prognostic marker.

**Conclusions:**

JS-K induces autophagy-dependent ferroptosis in bladder cancer cells and significantly suppresses tumor progression. Targeting the autophagy–ferroptosis axis offers a novel therapeutic strategy for bladder cancer treatment.

## Introduction

Bladder cancer is one of the most common urological malignancies, with urothelial carcinoma representing the predominant histological subtype, followed by squamous cell carcinoma and adenocarcinoma [1–3]. Standard therapeutic approaches include transurethral resection, intravesical chemotherapy, radical cystectomy combined with pelvic lymph node dissection, and urinary diversion [4]. Although combination chemotherapy provides survival benefits [5], its efficacy is limited by substantial toxicity, and the median survival of patients with advanced bladder cancer remains ∼9 months [6, 7].

In recent years, ion-regulated forms of cell death have garnered increasing attention for their relevance to cancer therapy [8, 9]. Ferroptosis, a distinct form of programmed cell death, is characterized by iron-dependent lipid peroxidation, excessive accumulation of reactive oxygen species (ROS), and dysregulated iron homeostasis [10, 11]. It is controlled by metabolic pathways involved in iron regulation, amino acid metabolism, and lipid oxidation, and can be initiated through both exogenous (transporter-mediated) and endogenous (enzyme-dependent) mechanisms [12]. Exogenous triggers include inhibition of the cystine/glutamate antiporter system (system Xc⁻) or upregulation of iron transport proteins such as transferrin and lactotransferrin. Endogenously, ferroptosis can be triggered by impaired antioxidant defenses—most notably through inhibition of glutathione peroxidase 4 (GPX4), a central regulator of cellular redox balance [12, 13].

JS-K is a nitric oxide (NO)-releasing prodrug developed by the US National Cancer Institute, and has demonstrated anti-tumor effects in several cancers, including liver, lung, ovarian, and bladder cancers [14–18]. JS-K has previously been shown to induce apoptosis in lung cancer cells through glutathione (GSH) depletion, mitochondrial oxidative stress, and cytochrome c release [16]. GSH depletion, ROS accumulation, and disrupted antioxidant defenses are also key initiators of ferroptosis. These overlapping upstream events suggest that JS-K engages redox vulnerabilities broadly, but the execution pathway—apoptosis versus ferroptosis—may depend on cellular context and iron/lipid metabolism.

Importantly, cancer cells appear to exhibit heightened sensitivity to ferroptosis compared with normal cells, highlighting its therapeutic potential [19–22]. While morphologically, biochemically, and genetically distinct from apoptosis, necrosis, and autophagy, ferroptosis is increasingly recognized to intersect with autophagy [23]. In particular, autophagy has been shown to promote iron-dependent cell death through selective degradation of ferritin (ferritinophagy), giving rise to the concept of autophagy-dependent ferroptosis [24]. In this study, we investigated the anti-tumor effects of JS-K in bladder cancer and examined the role of autophagy-dependent ferroptosis as a potential underlying mechanism.

## Materials and methods

### Cell-based assays to evaluate JS-K–induced ferroptosis and autophagy

Human bladder cancer cell lines T24 and UM-UC-3 were obtained from the Cell Bank of the Chinese Academy of Sciences (Shanghai, China). T24 cells were cultured in RPMI-1640 medium (GIBCO, USA), and UM-UC-3 cells were maintained in DMEM (GIBCO, USA), each supplemented with 10% fetal bovine serum (GIBCO, USA) and 1% penicillin–streptomycin (Solarbio, China). All cultures were incubated at 37 °C in a humidified atmosphere containing 5% CO₂. For the knock down of *LC3B*, shRNA targeting *LC3B* (5′-GCTAGAGAGATCTCCCTAAGA-3′) was synthesized by GenePharma (Shanghai, China) and cloned into lentiviral vectors (lentivirus viral titer was 1:250). Bladder cancer cells were transfected with *LC3B* shRNA or negative control shRNA (5′-TTCTCCGAACGTGTCACGT-3′). Western blot analysis was performed to detect the protein expression levels of both LC3B and LC3A. UM-UC-3 cells were transfected using Chemifect reagent (6 h prior to puromycin selection). After 48 h, the medium was replaced, and stably transfected clones were selected using puromycin (2 μg/ml) for 2 weeks. To measure the effects of JS-K on cell proliferation, T24 and UM-UC-3 cells were seeded in 96-well plates at a density of 3 × 10³ cells/well and cultured to ∼60% confluence. Cells were treated with 0.1% dimethyl sulfoxide (DMSO), JS-K, or JS-K in combination with ferrostatin-1 (Fer-1 1 μM), chloroquine (CQ, 5 μM), Z-VAD (40 μM), or necrostatin (Nec-1 10 μM) for 24 or 48 h. At each time point, 10 μl of CCK-8 reagent was added to each well and incubated for 2 h. Absorbance was measured at 450 nm using a microplate reader (EnSpire 2300 Multilabel Reader, PerkinElmer, USA). CCK8 assay was performed in triplicates, background absorbance controls included (medium + reagent, no cells).

### Reagents and antibodies

Anti-JS-K antibody (#SC-211683A, Santa Cruz Biotechnology, Dallas, TX, USA) was dissolved in DMSO to a stock concentration of 5 mM and stored at −80 °C. Primary antibodies against phospho-histone, GPX4 (#ab125066, 1:1000), transferrin receptor 1 (TFR1, #ab214039, 1:1000), ferritin heavy chain 1 (FTH1, #ab75973, 1:1000), solute carrier family 7 member 11 (SLC7A11, #ab307601, 1:1000), and LC3 (#ab192890, 1:1000) were purchased from Abcam (Cambridge, UK). MDA (#S0131S) and ROS (#S0033S) detection kits, as well as horseradish peroxidase-conjugated secondary antibodies (#A0208), were purchased from Beyotime Biotechnology (Shanghai, China). Cell Counting Kit-8 (CCK-8, #K009-500) was obtained from Zeta Life (NJ, USA). FerroOrange (#F374) was purchased from DOJINDO Laboratories (Kumamoto, Japan). Z-VAD-FMK (#HY-16658B), necrostatin-1 (Nec-1, #HY-15760), and ferrostatin-1 (Fer-1, #HY-100579) were obtained from MedChemExpress (NJ, USA)002E.

### Electron microscopy

Sapphire wafers were placed upright in 96-well plates, and T24 cells in logarithmic phase were seeded on the sapphire surface at 5000 cells/well in 100 μl medium. Following cell attachment, JS-K was added, and cells were incubated for 24 h. Sapphire wafers were then removed, fixed (2.5% glutaraldehyde in 0.1 M phosphate buffer, pH 7.4; 12 h; 4 °C). Ultrastructural imaging was performed using a transmission electron microscope operated at an accelerating voltage of 80 kV acquired across several magnification ranges (2500×, 8000×, 30 000×, and 60 000×) to visualize both overall cellular architecture and fine mitochondrial details. More than 10 fields were analyzed.

### Lipid peroxidation assay

T24 cells were treated with 1, 2, and 5 µM of JS-K for 24 h and washed three times with PBS. Cells were homogenized in RIPA buffer containing PMSF (100:1), incubated on ice for 15 min, sonicated, and centrifuged at 12 000 rpm for 20 min at 4 °C. Protein concentrations were determined using a bicinchoninic acid (BCA) assay. To assess lipid peroxidation, cell lysates were processed using a thiobarbituric acid reactive substances assay, in which malondialdehyde (MDA), a major byproduct of lipid peroxidation, reacts with thiobarbituric acid (TBA) under heating to form an MDA–TBA adduct. Samples were heated for 15 min, cooled to room temperature in a water bath, and centrifuged at 1000 g for 10 min. Lipid peroxidation was quantified by measuring absorbance at 532 nm with a microplate reader.

### Reactive oxygen species measurement

T24 and UM-UC-3 cells were seeded in six-well plates and treated with increasing concentrations (1, 2, and 5 µM) of JS-K for 24 h. After incubation, cells were collected and stained with the ROS-sensitive probe DCFH-DA (2′,7′-dichlorodihydrofluorescein diacetate) for 20 min. Within cells, DCFH-DA is deacetylated to the non-fluorescent DCFH, which is oxidized by ROS to form the fluorescent compound DCF. After washing to remove excess probe, cells were resuspended in serum-free medium, and fluorescence was quantified by flow cytometry or fluorescence microscopy (excitation 488 nm, emission 525 nm) as a measure of intracellular ROS.

### Intracellular iron measurement

T24 and UM-UC-3 cells were seeded into confocal culture dishes and treated with 1, 2, and 5 µM JS-K for 24 h. After medium removal, cells were rinsed thoroughly with serum-free medium and incubated with FerroOrange working solution for 30 min. FerroOrange is a cell-permeable fluorescent probe that selectively detects labile ferrous iron (Fe²⁺), the redox-active iron pool that drives lipid peroxidation during ferroptosis. Upon binding Fe²⁺, the probe exhibits enhanced fluorescence, which was visualized using an Olympus FV3000 laser confocal microscope.

### Detection of ferroptosis regulators, autophagy, and iron metabolism markers

Cells were treated with various concentrations of JS-K for 24 h, washed with cold phosphate-buffered saline (PBS), and lysed in RIPA buffer (Beyotime, China) containing 1 mM PMSF. Equal amounts of protein (∼20 μg) were separated on 12% SDS-PAGE gels and transferred to polyvinylidene difluoride (PVDF) membranes (Millipore, USA). Membranes were blocked with 5% non-fat milk, incubated with primary antibodies at 4 °C overnight, and then with secondary antibodies (HRP). Protein bands were visualized, and band intensity was quantified by ImageJ; analyzed to quantify relative protein expression.

### *In vivo* evaluation of JS-K anti-tumor effects in bladder cancer xenografts

Female BALB/c nude mice (4 weeks old) were purchased from the Southern Medical University Laboratory Animal Center. Mice were subcutaneously injected with 5 × 10⁶ wild-type or *LC3B*-knockdown UM-UC-3 cells. When tumors reached 50 mm³, mice were randomized into 0.9% saline or JS-K (20 mg/kg) groups. Treatments were administered via tail vein injection three times per week for 5 weeks. Tumor volumes were measured thrice weekly and calculated as (length × width²)/2. Relative tumor volume (RTV) was calculated by dividing by the baseline tumor volume at the start of treatment. At the end of the study, mice were euthanized and tumor tissues collected for analysis.

### Histological and immunohistochemical detection of autophagy–ferroptosis regulators in tumor tissues

Tumors were fixed in 10% formalin overnight, paraffin-embedded, and sectioned (4 μm). Hematoxylin and eosin (H&E) staining was performed using standard procedures. For immunohistochemistry (IHC), antigen retrieval was performed using citrate antigen retrieval solution. Sections were incubated with primary antibodies against LC3A/B (Cell Signaling Technology), GPX4, SLC7A11, and P62 (all from Abcam) at 4 °C overnight, and then with secondary antibodies (HRP), chromogen used DAB. The intensity and percentage of positive staining were quantified using Image-Pro Plus software (v6.0), and a total expression score was calculated.

### Multi-omics analysis of *LC3B* and ferroptosis pathways in bladder cancer

Bulk RNA-seq data of 402 bladder cancer samples were retrieved from The Cancer Genome Atlas (TCGA). Gene expression values were processed as log₂ (FPKM + 1) and accompanied by survival information. Ferroptosis-related genes were obtained from the KEGG pathway hsa04216. Pearson correlation analysis was used to assess associations between *LC3B* expression and ferroptosis-related genes, with correlations having |*r*| > 0.2 considered significant. Survival analyses were performed using the survival R package, and prognostic ferroptosis-related genes were identified using the random SurvivalForest package. Single-cell RNA-seq data were obtained from the Gene Expression Omnibus (accession number GSE222315). The data were processed and normalized using the Seurat package, with quality-control thresholds set at cells expressing 200–6000 genes and <10% mitochondrial gene content. Cell types were identified based on canonical marker genes. Bayesian networks were constructed with the bnlearn package, and structural equation modeling was conducted using lavaan to explore relationships between *LC3B* and ferroptosis-related genes at the single-cell level. Immune cell composition was estimated using CIBERSORT with the LM22 reference matrix. Ferroptosis-related genes that showed significant correlations with *LC3B* were subjected to OncoPrint visualization using the cBioPortal platform. Multiple-testing correction was performed using the Benjamini–Hochberg false discovery rate method, with an adjusted *P* < 0.05 considered significant.

### Statistical analysis

Statistical analyses were conducted using R version 4.4.2. Data are presented as mean ± standard deviation (SD). One-way analysis of variance (ANOVA) was used for multi-group comparisons, followed by the least significant difference test for pairwise comparisons. Two-way ANOVA testing followed by Tukey’s post-hoc testing with adjustment for multiple comparisons when indicated. *P*-value < 0.05 was considered statistically significant.

## Results

### JS-K induces autophagy-dependent ferroptosis in bladder cancer cells via the LC3B autophagic pathway

**Figure 1:**
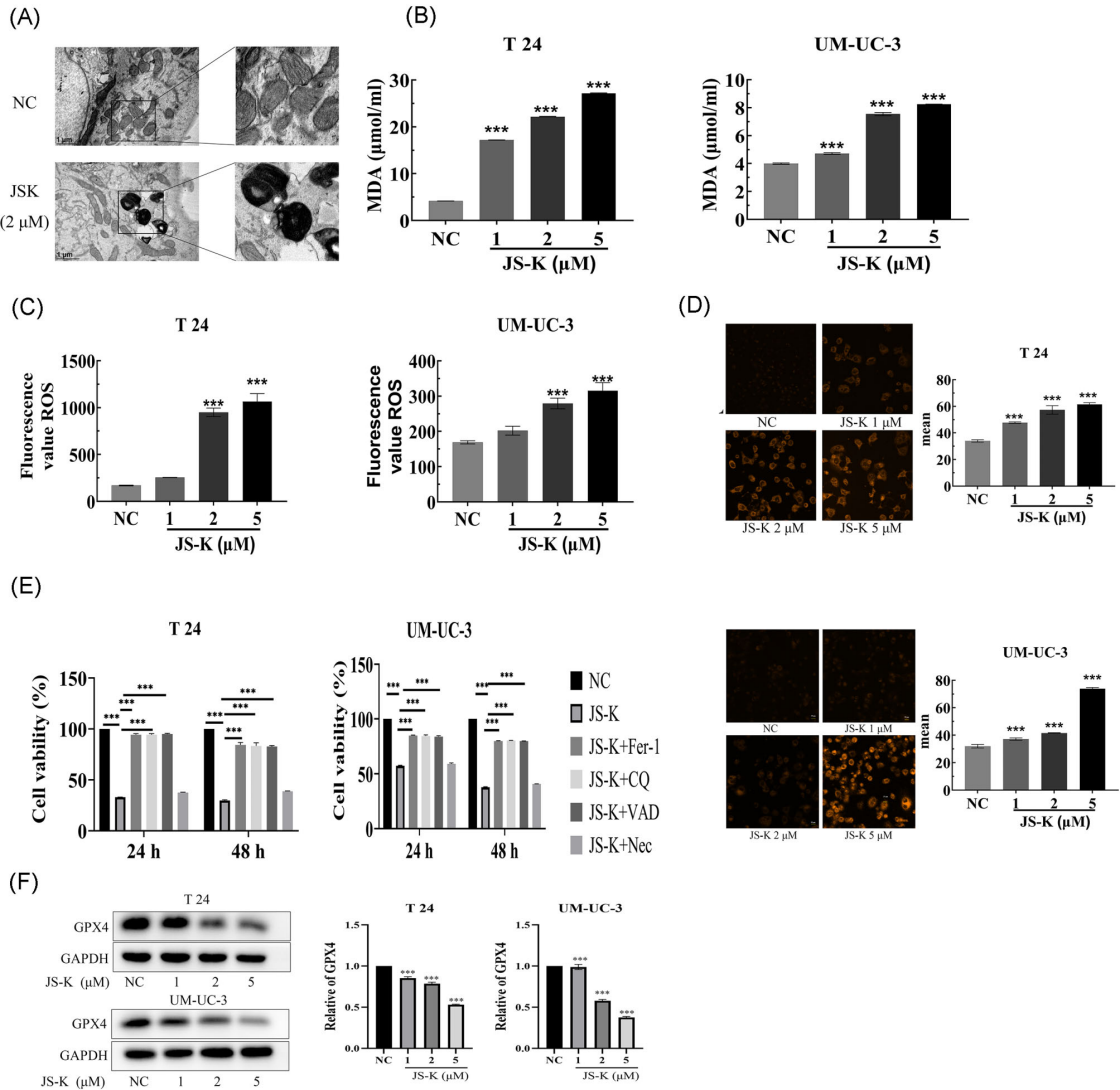
JS-K induces ferroptosis in bladder cancer cells. (A) Electron micrographs of T24 bladder cancer cells in control and after JS-K treatment. Mitochondrial shrinkage and increased membrane density were evident after JS-K treatment. Dose-dependent effects of JS-K on (B) intracellular lipid peroxidation levels; (C) intracellular reactive oxygen species levels; (D) dose-dependent increase in mean intracellular iron levels (FerroOrange) with JS-K treatment; (E) Fer-1, CQ, VAD, and Nec-1 levels; (two-way ANOVA with treatment and time (24 h vs. 48 h) as factors followed by Bonferroni’s multiple comparisons). A *P*-value < 0.05 was considered statistically significant. ***P < 0.001. Data are presented as the mean standard deviation (SD), *n* = 3 and (F) GPX4 protein expression level in T24 and UM-UC-3 bladder cancer cells. One-way analysis of variance was used to compare multi-group differences, and the Bonferroni method was used for the statistical analysis of pairwise comparisons. ***P < 0.001. Data are presented as the mean ± standard deviation (SD), *n* = 3.

To investigate the effect of JS-K on ferroptosis, T24 bladder cancer cells were treated with 2 μM JS-K for 24 h. Ultrastructural alterations by transmission electron microscopy showed that JS-K–treated cells exhibited mitochondrial shrinkage and increased membrane density, hallmark features of ferroptosis, compared with untreated controls (Fig. 1A). In both T24 and UM-UC-3 cells, JS-K dose-dependently increased lipid peroxidation, as indicated by elevated intracellular MDA measured using the thiobarbituric acid assay; enhanced ROS production, detected by oxidation of the DCFH-DA probe to fluorescent DCF; and increased intracellular iron levels, quantified using the FerroOrange probe, which selectively detects labile Fe²⁺ in live cells (Fig. 1B–D). Concomitantly, JS-K treatment led to a marked downregulation of GPX4, a key ferroptosis suppressor (Fig. 1F). These cytotoxic effects were alleviated by pharmacological inhibitors of ferroptosis (1 μM Fer-1), autophagy (5 μM CQ), apoptosis (40 μM Z-VAD), and necroptosis (10 μM Nec-1), indicating that JS-K triggers multiple regulated cell death pathways (Fig. 1E). JS-K did not induce necroptotic cell death since blocking necroptosis with Nec-1 did not protect cells from JS-K toxicity. To examine the temporal relationship between autophagy and ferroptosis, UM-UC-3 cells were treated with JS-K and analyzed at multiple time points. LC3B expression increased as early as 3 h and remained elevated at 6 and 12 h, while p62 expression decreased over the same period, consistent with early activation of autophagy. In contrast, ROS and MDA levels were unchanged at 3 h but increased significantly at 6, 12, and 24 h after JS-K treatment. These findings suggest that autophagy is induced at an early stage following JS-K exposure and precedes the accumulation of oxidative stress and lipid peroxidation, supporting an autophagy-dependent ferroptotic mechanism (Supplementary Fig. 1). To further assess autophagic processing, LC3 turnover was examined in UM-UC-3 cells treated with JS-K in the presence or absence of CQ. LC3-I (the cytosolic, non-lipidated form of LC3) levels were not significantly altered by JS-K, CQ, or the combination treatment. In contrast, LC3-II (the membrane-bound, lapidated form) levels increased after JS-K treatment and were further elevated by CQ alone and by JS-K + CQ, consistent with enhanced LC3 processing and accumulation of autophagosomes (Supplementary Fig. 2).

**Figure 2:**
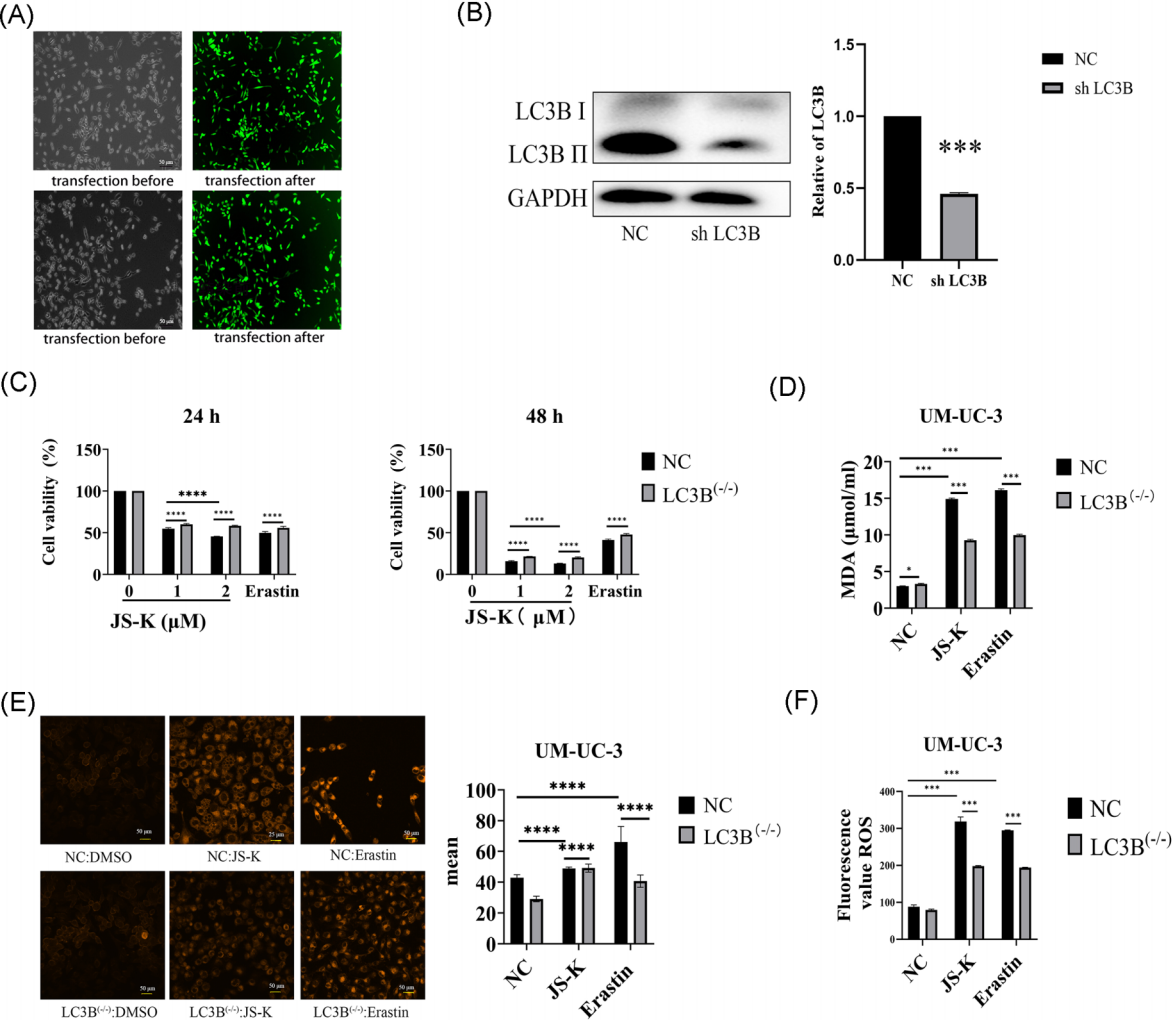
*LC3B* knockdown attenuates JS-K– and erastin-induced ferroptosis in bladder cancer cells. (A) Transfection efficiency in the negative control (NC) and *LC3B* knockdown (UM-UC-3^LC3B–/–) groups was monitored by GFP fluorescence encoded in the shRNA vector; (B) LC3B protein expression was significantly reduced in LC3B ^(−/−)^ cells compared to NC cells. ***P < 0.001. Data are presented as the mean standard deviation (SD), *n* = 3; (C) JS-K and erastin reduced cell viability in a time-dependent manner. LC3B-deficient cells exhibited higher survival under 1 µM and 2 µM JS-K treatments, indicating that JS-K engages autophagy via LC3B to promote ferroptotic cell death. Erastin treatment showed a similar effect to JS-K where LC3B-deficient cells had higher viability; (D) JS-K and erastin significantly increased intracellular MDA levels, while LC3B knockdown reduced MDA accumulation, indicating that LC3B-mediated autophagy promotes lipid peroxidation during ferroptosis; (E) JS-K and erastin increased mean FerroOrange fluorescence. LC3B^(−/−)^ cells showed significantly less intracellular iron accumulation; (F) JS-K and erastin increased ROS levels. LC3B^(−/−)^ cells showed significantly less ROS accumulation. Two-way analysis of variance (ANOVA) testing with a *P*-value threshold of 0.05, followed by Tukey’s post-hoc testing with adjustment for multiple comparisons when indicated. ***P < 0.001, ****P < 0.0001. Data are presented as the mean ± standard deviation (SD), *n* = 3.

**Figure 3:**
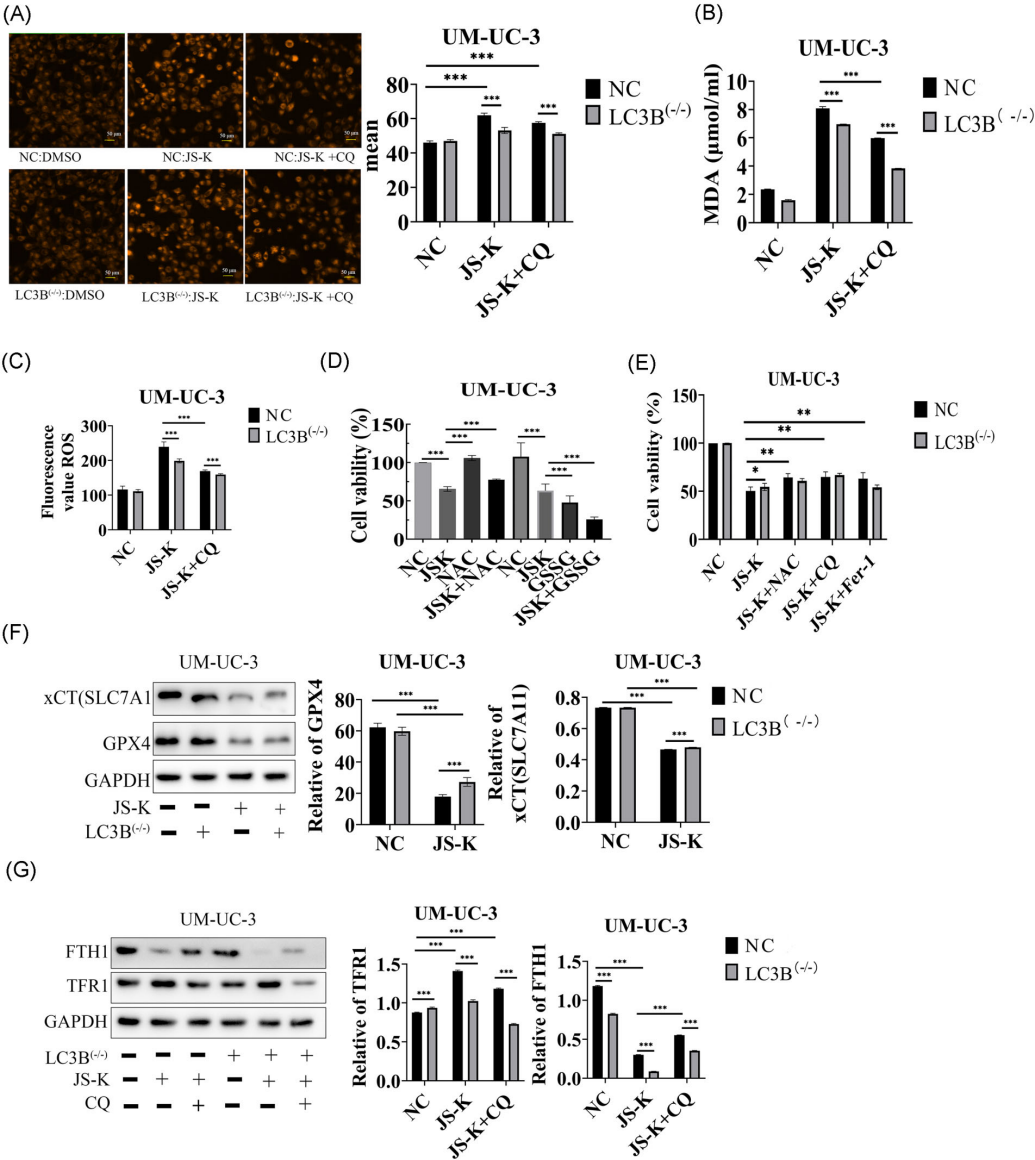
Autophagy and redox balance modulate JS-K–induced ferroptosis and regulation of ferroptosis-related proteins. (A) Intracellular iron levels measured with the FerroOrange probe increased after JS-K treatment, but this effect was attenuated in LC3B-deficient cells. (B) Malondialdehyde (MDA) levels were significantly elevated after JS-K treatment and reduced in LC3B-deficient cells. MDA levels were further reduced by chloroquine (CQ) treatment with JS-K. CQ reduced peroxidation even further in LC3B-deficient cells. (C) Reactive oxygen species (ROS) levels measured with the DCFH-DA probe increased after JS-K treatment, but were significantly reduced in LC3B-deficient cells and with CQ. (D) Cell viability was significantly decreased by JS-K treatment, rescued by N-acetylcysteine (NAC), and further reduced by the oxidizing agent GSSG. (E) LC3B-deficient cells showed higher survival than controls after JS-K treatment; however, no differences were observed between groups under JS-K + NAC, JS-K + CQ, or JS-K + Fer-1 indicating that these inhibitors target the same cell death pathways as LC3B. (F) JS-K treatment downregulated xCT (SLC7A11) and GPX4 protein expression, xCT (SLC7A11) and GPX4 protein expression were rescued in LC3B-deficient cells treated with JS-K but still significantly reduced compared to no treatment. (G) JS-K increased TFR1 expression in wild-type cells but not in LC3B−/− cells, and a similar pattern was observed with JS-K + CQ. For FTH1, JS-K reduced its expression in wild-type cells and caused an even greater reduction in LC3B−/− cells. JS-K + CQ also lowered FTH1 compared with untreated controls, though levels were higher than with JS-K alone; however, LC3B−/− cells still showed a more pronounced decrease than wild-type cells under JS-K + CQ treatment. Two-way analysis of variance (ANOVA) (genotype × treatment) testing with a *P*-value threshold of 0.05, followed by Tukey’s post-hoc testing with adjustment for multiple comparisons when indicated. ***P < 0.001, ****P < 0.0001. Data are presented as the mean ± standard deviation (SD), *n* = 3.

To determine the role of autophagy, we silenced *LC3B* expression in UM-UC-3 cells using an shRNA construct (UM-UC-3^LC3B–/–). Knockdown efficiency was confirmed by fluorescence microscopy and western blotting (Fig. 2A, B). JS-K and erastin reduced cell viability in a time-dependent manner, but LC3B-deficient cells displayed higher survival compared with parental controls (Fig. 2C). *LC3B* knockdown reversed JS-K–induced increases in iron, MDA, and ROS (Fig. 2D–F). Similarly, CQ, an autophagy inhibitor, mitigated JS-K cytotoxicity (Fig. 3A–C). The antioxidant N-acetylcysteine (NAC) also rescued cell viability in both UM-UC-3 and UM-UC-3^LC3B–/– cells, whereas the oxidizing agent GSSG enhanced JS-K lethality (Fig. 3D, E). At the molecular level, knockdown of *LC3B* partially but significantly restored GPX4 and xCT expression that was suppressed by JS-K treatment, suggesting that autophagy contributes to the downregulation of these ferroptosis-protective proteins (Fig. 3F). JS-K also increased TFR1, which promotes iron uptake, and decreased FTH1, responsible for iron storage, thereby favoring expansion of the labile iron pool and enhanced lipid peroxidation. These effects were blunted in LC3B-deficient cells, indicating that LC3B-mediated autophagy facilitates iron dyshomeostasis during JS-K–induced ferroptosis. Consistently, both JS-K and JS-K + CQ significantly increased TFR1 compared with untreated controls, with no difference between the two treatments. In contrast, FTH1 expression was significantly higher in JS-K + CQ–treated cells compared with JS-K alone, although both remained reduced relative to untreated controls. Importantly, *LC3B* knockdown further reduced TFR1 and FTH1 under JS-K + CQ treatment compared with wild-type cells (Fig. 3G).

Notably, *LC3B* knockout further exacerbated JS-K–induced downregulation of FTH1, an observation that initially appears counterintuitive to the role of LC3B-mediated autophagy in iron metabolism. We speculate that this effect arises from compensatory activation of alternative autophagic pathways and iron metabolic reprogramming in the absence of LC3B. While CQ only partially blocks autophagic flux by inhibiting lysosomal fusion, complete loss of LC3B triggers robust compensation, potentially involving LC3A/LC3C-dependent autophagy or NCOA4-mediated ferritinophagy. To define the specific contribution of NCOA4 to this process, we employed gene silencing strategies. *NCOA4* knockdown significantly attenuated JS-K-induced cytotoxicity and partially mitigated the associated surge in lipid peroxidation and intracellular iron overload. While basal and JS-K-treated ROS levels were suppressed in NCOA4-deficient cells suggesting a role for NCOA4 in sustaining an oxidative milieu, the lack of a significant ROS elevation in the JS-K-treated control group (siNC + JS-K) relative to untreated controls precludes a definitive conclusion regarding NCOA4 as the mediator of JS-K-driven ROS generation (Supplementary Fig. 3). Collectively, these findings establish NCOA4-mediated ferritinophagy as a pivotal downstream effector in JS-K-induced ferroptosis.

### JS-K suppresses tumor growth *in vivo* via autophagy–ferroptosis coupling

**Figure 4:**
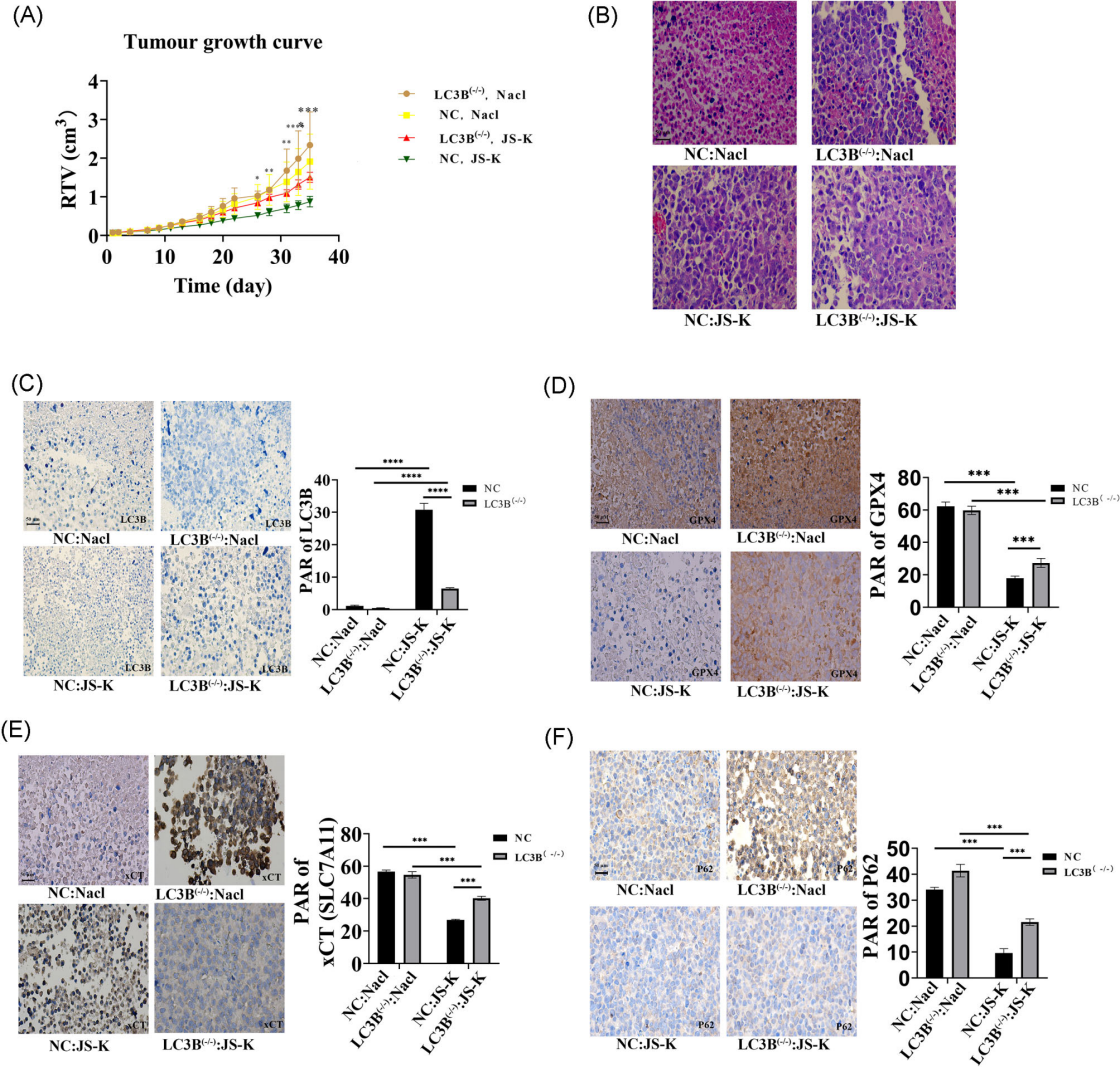
JS-K inhibits bladder tumor growth *in vivo*. (A) Significant decrease in relative tumor volume (RTV) after JS-K treatment from 26 days. (B) HE staining of tumor tissue of WT and LC3B−/− mice after JS-K treatment. (C) LC3B expression was low in saline-treated UM-UC-3 tumors but significantly increased following JS-K treatment. Induction of LC3B by JS-K was weaker in LC3B-deficient tumors. (D) GPX4 was highly expressed in saline-treated tumors and significantly downregulated after JS-K treatment in both UM-UC-3 and LC3B-deficient tumors. The reduction was greater in WT tumors. (E) xCT expression was high under saline conditions but decreased significantly with JS-K treatment. The reduction was more pronounced in WT tumors than in LC3B-deficient tumors. (F) p62 levels were elevated in LC3B-deficient tumors under saline conditions, consistent with impaired autophagic flux. JS-K treatment reduced p62 expression in both WT and LC3B-deficient tumors, with a greater decrease observed in UM-UC-3 tumors. Two-way analysis of variance (ANOVA) testing with a *P*-value threshold of .05, followed by Tukey’s post-hoc testing with adjustment for multiple comparisons when indicated. ***P < 0.001, ****P < 0.0001. Data are presented as the mean ± standard deviation (SD), *n* = 3.

To validate the *in vitro* findings, we established xenograft models using UM-UC-3 and UM-UC-3^LC3B–/– cells. Mice were treated with either JS-K or vehicle control. JS-K significantly inhibited the growth of wild-type xenografts but produced minimal growth inhibition in LC3B-deficient tumors (Fig. 4A). Tumor tissues from JS-K–treated mice showed reduced expression of GPX4 and SLC7A11 (Fig. 4B). HE staining also showed reduced tumor cell density and increased structural degeneration in JS-K–treated wild-type tumors compared with controls. LC3B expression was very low in saline-treated UM-UC-3 tumors but was significantly upregulated following JS-K treatment. In LC3B-deficient tumors, JS-K also induced LC3B expression, though to a much lesser extent due to residual LC3B after knockdown (Fig. 4C, F). This indicates that JS-K activates autophagy in wild-type tumors; in LC3B-deficient tumors, the induction is blunted suggesting that autophagy is part of JS-K’s mechanism. GPX4 and SLC7A11 were both highly expressed in saline-treated UM-UC-3 and UM-UC-3^LC3B–/– tumors, with no difference between the two groups. JS-K treatment significantly decreased GPX4 and SLC7A11 in both tumor types, but the reduction was more pronounced in UM-UC-3 than in UM-UC-3^LC3B–/– tumors (Fig. 4D, E). This is in accordance with our finding that JS-K suppresses ferroptosis-protective proteins. This suppression is stronger in WT tumors than in LC3B-deficient tumors. Our data show that LC3B enhances ferroptotic vulnerability. Expression of the autophagy substrate p62 was elevated in UM-UC-3^LC3B–/– tumors compared with wild-type tumors under saline conditions, consistent with impaired autophagic flux. JS-K significantly reduced p62 expression in both tumor types, with a greater reduction in UM-UC-3 tumors than in UM-UC-3^LC3B–/– tumors (Fig. 4F). Our data suggest that JS-K drives ferroptosis by activating autophagy, suppressing ferroptosis defenses, and reducing p62; these effects are attenuated when LC3B is deficient, confirming that autophagy amplifies JS-K–induced ferroptosis *in vivo*.

### *LC3B* correlates with ferroptosis gene expression and bladder cancer prognosis

**Figure 5:**
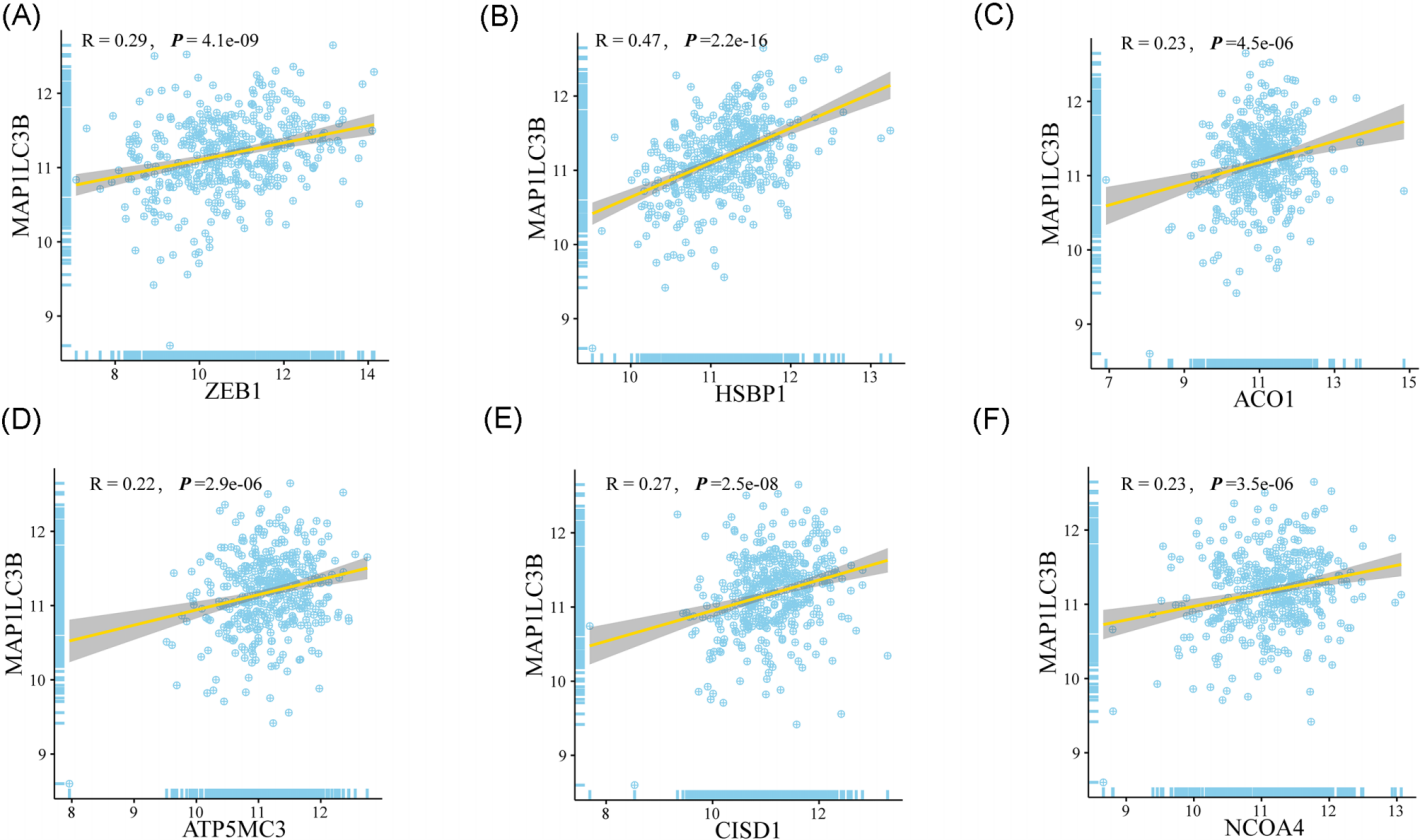
Bulk RNA-seq analysis of bladder cancer samples reveals correlation between *LC3B* and ferroptosis-related genes via transcriptome data. (A) *ZEB1* expression is positively correlated with *LC3B* expression (correlation coefficient = 0.29); (B) *HSBP1* expression is positively correlated with *LC3B* expression (correlation coefficient = 0.47); (C) *ACO1* expression is positively correlated with *LC3B* expression (correlation coefficient = 0.23); (D) *ATP5MC3* expression is positively correlated with *LC3B* expression (correlation coefficient = 0.22); (E) *CISD1* expression is positively correlated with *LC3B* expression (correlation coefficient = 0.27); (F) *NCOA4* expression is positively correlated with *LC3B* expression (correlation coefficient = 0.23).

**Figure 6:**
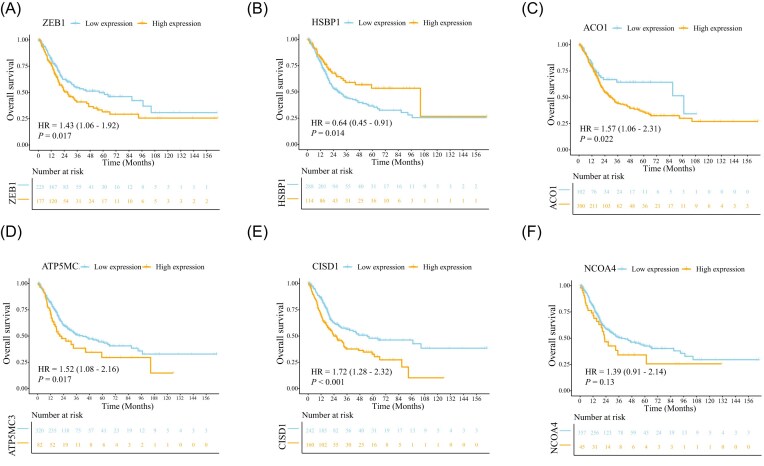
The expression of six ferroptosis-associated genes can affect the prognosis of bladder cancer. Kaplan–Meier survival analysis showed that high expression of *ZEB1, ACO1, ATP5MC3, CISD1*, and *NCOA4* was associated with poorer overall survival, whereas low expression of *HSBP1* was linked to worse prognosis.

Bulk RNA-seq analysis of bladder cancer samples from TCGA revealed that *LC3B* expression was positively correlated with multiple ferroptosis-related genes. Specifically, *LC3B* showed significant correlations with *ZEB1* (*r* = 0.29), *HSBP1* (*r* = 0.47), *ACO1* (*r* = 0.23), *ATP5MC3* (*r* = 0.22), *CISD1* (*r* = 0.27), and *NCOA4* (*r* = 0.23) (Fig. 5A–F). Among these, *HSBP1* displayed the strongest association with *LC3B*. These correlations suggest that autophagy, as reflected by *LC3B* expression, is linked to pathways that regulate ferroptosis sensitivity, including iron metabolism (*ACO1, NCOA4, CISD1*), mitochondrial function (*ATP5MC3, CISD1*), and stress response (*HSBP1, ZEB1*). The correlation with *NCOA4*, a mediator of ferritinophagy, supports our experimental findings that autophagy contributes to iron-dependent lipid peroxidation during JS-K–induced ferroptosis. Kaplan–Meier survival analysis demonstrated that the expression of six ferroptosis-related genes with prognostic relevance was significantly associated with overall survival in bladder cancer patients. High expression of *ZEB1, ACO1, ATP5MC3, CISD1*, and *NCOA4* was each linked to poorer prognosis (Fig. 6A, C–F). In contrast, low expression of *HSBP1* was associated with reduced survival (Fig. 6B). These findings indicate that dysregulation of ferroptosis-associated genes can stratify bladder cancer patients by prognosis. Genes such as *NCOA4* and *CISD1*, which regulate iron homeostasis and mitochondrial function, may drive tumor progression when overexpressed, whereas *HSBP1* may act in a protective capacity, with reduced expression impairing stress responses and correlating with worse outcomes. In addition to ferroptosis-related genes, we further evaluated the prognostic significance of *LC3B* expression. Kaplan–Meier analysis showed that *LC3B* exhibited a trend toward worse overall survival in the overall cohort, although this did not reach statistical significance (HR = 1.55, *P* = 0.056). Notably, subgroup analyses revealed marked heterogeneity in its prognostic impact. Elevated *LC3B* expression was significantly associated with poorer survival in patients with advanced tumor stage (stage IV; HR = 2.61, *P* = 0.008) and in female patients (HR = 2.28, *P* = 0.007), while showing variable trends across other clinical subgroups (Supplementary Fig. 4). These findings indicate that the prognostic value of *LC3B* is context-dependent and may become more pronounced under specific clinical conditions.

**Figure 7:**
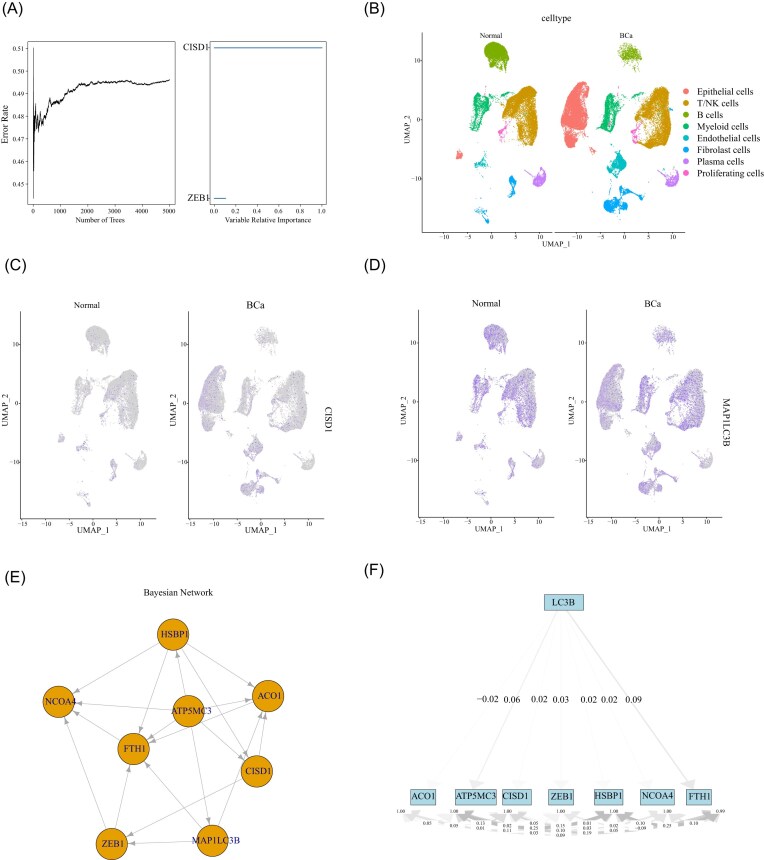
Correlation between *LC3B* and ferroptosis-related genes validated using single-cell sequencing data. (A) Random forest algorithm identified CISD1 as having the greatest impact on the prognosis of bladder cancer patients. (B) Cell type classification in bladder cancer single-cell RNA-seq data (GSE222315) using Uniform Manifold Approximation and Projection (UMAP) plot. (C) *CISD1* is primarily expressed in tumor epithelial cells. (D) *LC3B* expression shows no significant difference between normal and tumor tissues. (E) Bayesian network analysis reveals a close association between *LC3B* and ferroptosis-related genes. (F) Structural equation modeling validates the correlation between *LC3B* and ferroptosis-related genes at the single-cell level.

Tumor mutation profiling using cBioPortal revealed that *LC3B* amplification frequently co-occurred with *HSBP1* amplification in bladder cancer patients, although most cases did not harbor significant point mutations or deletions in these genes (Supplementary Fig. 5). This suggests that *LC3B* exerts its functional impact primarily through expression changes rather than genetic mutations. To explore the relationship between *LC3B* and the tumor immune microenvironment, we performed immune cell deconvolution using CIBERSORT. Heatmap analysis showed heterogeneous enrichment of immune cell subsets across bladder cancer samples (Supplementary Fig. 6A). Correlation analysis demonstrated that *LC3B* expression was negatively associated with activated dendritic cells (*r* = −0.20) and naive CD4⁺ T cells (*r* = −0.15), while positively correlated with M2 macrophages (*r* = 0.14) and resting memory CD4⁺ T cells (*r* = 0.13) (Supplementary Fig. 6B–E). These findings suggest that high *LC3B* expression is linked to an immunosuppressive tumor phenotype, characterized by reduced antigen-presenting activity and increased protumor macrophage infiltration. Random survival forest analysis identified *CISD1* as the most influential ferroptosis-related gene affecting bladder cancer prognosis (Fig. 7A). Using single-cell RNA-seq data (GSE222315), we performed UMAP clustering to classify cell types within bladder tumors (Fig. 7B). *CISD1* expression was enriched in tumor epithelial cells (Fig. 7C), consistent with its role in regulating iron metabolism within malignant compartments, while *LC3B* expression showed no significant difference between tumor and adjacent normal tissues (Fig. 7D). Bayesian network analysis revealed strong associations between *LC3B* and multiple ferroptosis-related genes (Fig. 7E), and structural equation modeling further validated these correlations at the single-cell level (Fig. 7F). Together, these analyses confirm that *LC3B* is functionally linked to ferroptosis-related genes in tumor epithelial cells and support its role as a mediator of ferroptosis in bladder cancer.

## Discussion

Most patients with advanced urothelial carcinoma of the bladder are not candidates for surgical resection and are typically treated with platinum-based combination chemotherapy. However, bladder tumors are prone to recurrence and frequently acquire resistance to chemotherapy, a major challenge that compromises the efficacy of conventional treatments [25]. While recent advances in intravesical therapy have provided new options for managing bladder cancer, limitations such as toxicity and incomplete efficacy still exist, highlighting the unmet clinical need for novel therapeutic strategies [26]. The molecular mechanisms underlying bladder cancer progression remain critical for the development of novel therapeutic interventions. In this study, we demonstrate that JS-K induces autophagy-dependent ferroptosis in bladder cancer cells, both *in vitro* and *in vivo*. Mechanistically, LC3B-mediated autophagy plays a pivotal role in facilitating JS-K–induced ferroptotic cell death. Consistent with this, JS-K triggered mitochondrial damage, lipid peroxidation, ROS accumulation, and iron overload, accompanied by downregulation of GPX4 and SLC7A11. These findings suggest that targeting the autophagy–ferroptosis axis may represent a promising therapeutic strategy for bladder cancer.

JS-K has been reported to exhibit anti-tumor activity in multiple malignancies. Liu *et al*.  demonstrated that JS-K induces apoptosis in hepatocellular carcinoma cells through PP2A activation, which contributed to the regulation of Bcl-2 family proteins [27]. Similarly, Zhao *et al*.  reported that JS-K suppresses antioxidant enzymes such as Cu/Zn-superoxide dismutase (SOD1) and catalase in gastric cancer cells, leading to ROS accumulation and cell death [28]. Song *et al*.  found that JS-K induces G₂/M cell cycle arrest and apoptosis in A549 and H460 lung cancer cells, potentially via activation of the p53/p21^WAF1/CIP1 and p27^KIP1 pathways [29].

In addition to its anti-tumor activity, prior preclinical studies suggest that JS-K is tolerable *in vivo* at therapeutically effective doses. In a non-small-cell lung cancer xenograft model, intravenous JS-K administered three times weekly significantly inhibited tumor growth without affecting body weight [16]. Similarly, in multiple myeloma xenograft models, repeated intravenous dosing produced marked tumor suppression and prolonged survival, and previous work cited therein reported no significant toxicity, including systemic hypotension, at the doses used [30]. In an ovarian cancer xenograft model, JS-K also significantly suppressed tumor growth without significant body weight loss and caused less liver injury than cisplatin [17]. Although these studies support the feasibility of JS-K as an experimental anticancer agent, comprehensive safety, pharmacokinetic, and toxicology studies will still be required before translational application. In bladder cancer, Qiu *et al*.  showed that JS-K induces ROS-dependent apoptosis, which can be reversed by NAC [14]. In our study, JS-K exhibited cytotoxic effects on bladder cancer cells, which were mitigated by inhibitors of apoptosis, autophagy, and ferroptosis, as well as by antioxidants. In contrast, oxidative stress enhanced the cytotoxicity of JS-K, implicating ROS accumulation as a central mechanism in its anti-tumor activity. Activating ferroptosis has emerged as a promising approach to overcome chemoresistance in cancer, particularly by circumventing traditional apoptotic pathways [31]. Several chemotherapeutic agents have been shown to trigger ferroptosis through mechanisms such as inhibition of the system Xc⁻ antiporter [32, 33], suppression or depletion of GPX4 [34], and upregulation of p53 [35]. Targeted therapies, such as fibroblast growth factor receptor (FGFR) inhibitors, have also shown promise in urothelial carcinoma, though not all patients benefit from these strategies [36]. Additionally, non-coding RNAs have been identified as critical regulators of bladder cancer progression and chemoresistance, further enriching the molecular regulatory network of this disease [37]. In our study, JS-K treatment caused mitochondrial damage, increased autophagosome formation, and enhanced lipid peroxidation, ROS generation, and iron accumulation in a dose-dependent manner. Downregulation of GPX4 further confirmed the induction of ferroptosis.

Autophagy-dependent ferroptosis offers a novel therapeutic avenue in bladder cancer. For example, Sun *et al*.  reported that FIN56 induces ferroptosis through autophagy-mediated degradation of GPX4 and acts synergistically with mTOR inhibitors to enhance tumor cell killing [38]. Accumulating evidence has linked endoplasmic reticulum stress pathways, especially the IRE1α axis, to the therapeutic response and ferroptosis regulation in bladder cancer [39], which aligns with our findings that autophagy (a key downstream event of ER stress) facilitates JS-K–driven ferroptosis. In our study, *LC3B* knockdown and CQ treatment reversed JS-K–induced increases in iron, ROS, and lipid peroxidation, demonstrating that autophagy facilitates JS-K–driven ferroptosis. Time-course analysis showed that LC3B upregulation and p62 reduction occurred at early time points after JS-K exposure, whereas ROS accumulation and MDA elevation became apparent later, indicating that autophagy activation precedes overt ferroptotic damage. Consistent with this, LC3-II turnover analysis showed increased LC3-II accumulation after JS-K treatment, which was further enhanced in the presence of CQ, supporting increased autophagic processing following JS-K exposure. To explore the downstream pathway linking autophagy to ferroptosis, we silenced NCOA4, a key mediator of ferritinophagy. *NCOA4* knockdown attenuated JS-K-induced iron accumulation and cytotoxicity, while only partially reducing lipid peroxidation, suggesting that ferritinophagy contributes to the ferroptotic response but may not fully account for all oxidative damage induced by JS-K. Our data suggest that JS-K-induced LC3B-dependent autophagy promotes ferroptosis at least in part through ferritinophagy-mediated iron dysregulation. *In vivo*, JS-K significantly suppressed tumor growth, but this effect was markedly reduced in LC3B-deficient xenografts, further confirming that LC3B-mediated autophagy amplifies ferroptotic cell death.

Beyond our experimental observations, analysis of TCGA data further revealed that the prognostic relevance of *LC3B* is not uniform across all patients but exhibits marked context-dependent heterogeneity. Although *LC3B* expression did not show a strong overall association with survival in the unstratified cohort, subgroup analyses demonstrated distinct patterns across clinical settings. Notably, elevated *LC3B* expression was significantly associated with worse prognosis in advanced tumor stages (stage IV) and in female patients, while showing variable trends in other subgroups. These findings suggest that the prognostic impact of *LC3B* is highly dependent on tumor context rather than acting as a universal biomarker. This heterogeneity may reflect differential activation of LC3B-mediated autophagy under specific tumor states or microenvironmental conditions. Given that autophagy is primarily regulated at the level of dynamic flux rather than static transcript abundance, the subgroup-specific differences observed here may better capture variations in functional autophagic activity that are not fully reflected by bulk expression levels [40]. Consistent with our experimental findings, such context-dependent activation of LC3B-associated autophagy may modulate ferroptosis sensitivity, thereby influencing tumor progression and clinical outcomes in bladder cancer [23]. In parallel, transcriptomic analysis revealed that *LC3B* expression correlates with multiple ferroptosis-related genes, including *CISD1, ACO1, NCOA4*, and *ATP5MC3* [41]. These associations support the concept that autophagic activity is coordinated with iron-handling and redox-regulatory pathways that shape ferroptosis susceptibility in bladder cancer. Among these genes, *CISD1* emerged as the most prognostically influential in our random survival forest analysis, despite only modest correlation with *LC3B*. This significance reflects its biological function: CISD1 restricts mitochondrial iron accumulation and prevents lipid peroxidation, thereby limiting ferroptotic cell death [42]. These findings further support a model in which LC3B functions as a state-dependent regulator of ferroptosis rather than a constitutively active pathway in bladder cancer.

CISD1 is known to inhibit ferroptosis by limiting mitochondrial iron accumulation and lipid peroxidation. However, in our analysis, high CISD1 expression was associated with poor prognosis. This apparent paradox may reflect a ferroptosis-resistant tumor phenotype. Tumors with high CISD1 expression may possess enhanced capacity to buffer iron-dependent oxidative damage, thereby promoting survival under stress conditions. Consistent with this, recent studies have shown that CISD1 is upregulated in multiple cancers, and its high expression correlates with poor clinical outcomes and immunosuppressive features [43, 44]. Moreover, ferroptosis resistance has been linked to tumor progression and reduced therapeutic sensitivity [45]. In this context, JS-K-induced ferroptosis may exert therapeutic effects by overcoming this intrinsic resistance, highlighting a potential therapeutic vulnerability in CISD1-high bladder cancers. Single-cell RNA-seq demonstrated that *CISD1* is enriched in malignant epithelial cells, suggesting that tumor-intrinsic ferroptosis resistance may contribute to disease progression. Consistent with this, high CISD1 expression was associated with poorer overall survival, indicating potential prognostic and therapeutic relevance.

Finally, JS-K altered multiple iron-regulatory proteins in ways consistent with ferroptosis induction. FTH1 catalyzes the oxidation of Fe²⁺ to Fe³⁺, facilitating iron storage, transport, and redox balance [46]. Ferritin light chain (FTL) contributes to ferritin stability and nucleation of iron cores. Both FTH1 and FTL can promote ferroptosis by increasing intracellular iron levels via autophagic mechanisms [47]. Kong *et al*.  demonstrated that flavitin induces ferroptosis in bladder cancer by downregulating FTH1 [48]. The cystine/glutamate antiporter subunit SLC7A11 is highly expressed in various cancers [49], promoting tumor growth by increasing cystine uptake, supporting GPX4 synthesis, and reducing oxidative stress [50]. Moreover, p53 has been shown to induce ferroptosis by repressing SLC7A11 [51]. Wang *et al*. found that HSPA5 mediates ferroptosis in bladder cancer through inhibition of the p53/SLC7A11/GPX4 axis [52]. In our study, JS-K reduced FTH1 expression while increasing TFR1, indicating altered iron handling during ferroptotic stress. Notably, the reduction in FTH1 was more pronounced in LC3B-deficient cells, suggesting that the effect of LC3B loss on ferritin regulation is more complex than a simple linear autophagy model and may reflect secondary alterations in iron handling during JS-K treatment. In contrast, TFR1 induction was attenuated in LC3B-deficient cells, indicating that LC3B-mediated autophagy contributes to selected components of JS-K-induced iron dysregulation. These findings suggest that genetic LC3B loss and pharmacological lysosomal inhibition do not affect iron metabolism identically, and that LC3B deficiency may trigger secondary compensatory changes in ferritin regulation beyond those produced by CQ alone. Additionally, JS-K reduced p62 levels more strongly in wild-type tumors, supporting enhanced autophagic flux in an LC3B-dependent manner. Beyond its direct effects on tumor cell death, ferroptosis has also been increasingly recognized to influence the tumor immune microenvironment. In our study, LC3B expression was associated with increased infiltration of M2 macrophages and resting memory CD4⁺ T cells, suggesting a more immunosuppressive tumor phenotype. Given that LC3B-mediated autophagy enhances ferroptotic cell death, these findings raise the possibility that autophagy–ferroptosis coupling may also shape immune contexture in bladder cancer. Emerging evidence indicates that ferroptosis can function as a form of immunogenic cell death, capable of releasing damage-associated molecular patterns and modulating immune cell recruitment and activation [53]. In this context, JS-K–induced ferroptosis may not only directly suppress tumor growth but also partially reprogram the immunosuppressive microenvironment toward a more responsive state. Although this hypothesis requires further experimental validation, it highlights a potential dual role of targeting the autophagy–ferroptosis axis in both tumor cell intrinsic and immune-mediated therapeutic responses.

## Conclusions

JS-K inhibits the growth of bladder cancer cells via an autophagy-dependent ferroptotic mechanism. We identified positive associations between autophagy-related *LC3B* and several ferroptosis-associated genes in bladder cancer, and highlighted *CISD1* as a prognostically relevant ferroptosis-related gene. These findings provide a mechanistic rationale and experimental foundation for further preclinical development of JS-K as a potential therapeutic agent in the treatment of bladder cancer.

## Ethical statement

Female BALB/c nude mice (SPF grade) were purchased from SiBeiFu (Beijing) Biotechnology Co., Ltd. All animal experiments were approved by the Laboratory Animal Ethics Committee of Affiliated Hospital of Guangdong Medical University (approval no. AHGDMU-LAC-B-202210-0059). The animal use license was SYXK (Yue) 2022-0286, valid for barrier-housed rats and mice. All animal procedures were performed in accordance with institutional and national guidelines for laboratory animal welfare and ethics.

## Supplementary Material

pbag012_Supplemental_File
