# Supplementary material for: JS-K induces autophagy-dependent ferroptosis in bladder cancer: a multimodal mechanistic and translational study
Source: Precis Clin Med. 2026 Apr 25;9(2):pbag012. doi: 10.1093/pcmedi/pbag012 (PMC13197665; doi:10.1093/pcmedi/pbag012)
Supplement: pbag012_Supplemental_File [file pbag012_supplemental_file.pptx]

## Slide 1
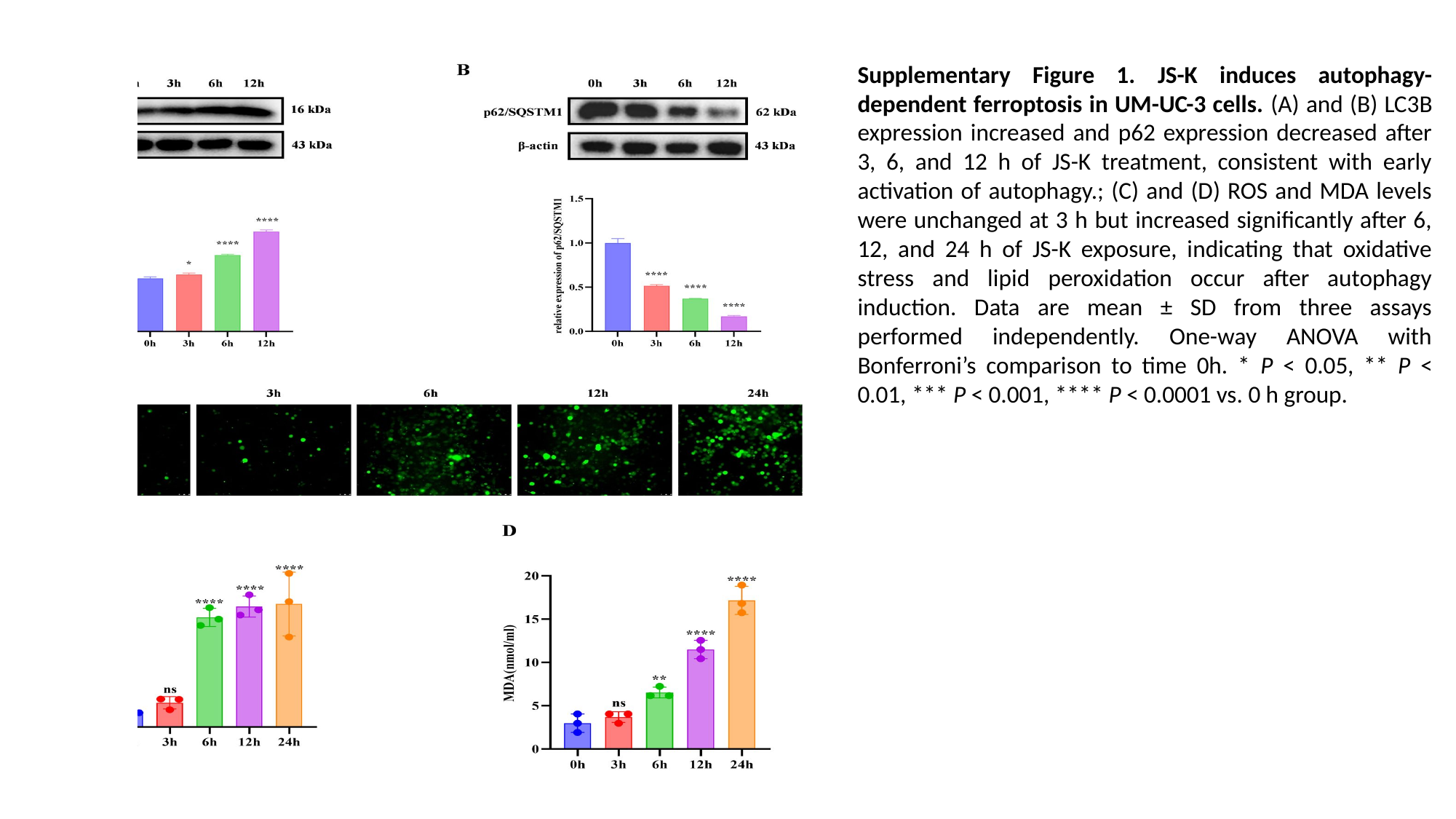

Supplementary Figure 1. JS-K induces autophagy-dependent ferroptosis in UM-UC-3 cells. (A) and (B) LC3B expression increased and p62 expression decreased after 3, 6, and 12 h of JS-K treatment, consistent with early activation of autophagy.; (C) and (D) ROS and MDA levels were unchanged at 3 h but increased significantly after 6, 12, and 24 h of JS-K exposure, indicating that oxidative stress and lipid peroxidation occur after autophagy induction. Data are mean ± SD from three assays performed independently. One-way ANOVA with Bonferroni’s comparison to time 0h. * P < 0.05, ** P < 0.01, *** P < 0.001, **** P < 0.0001 vs. 0 h group.

## Slide 2
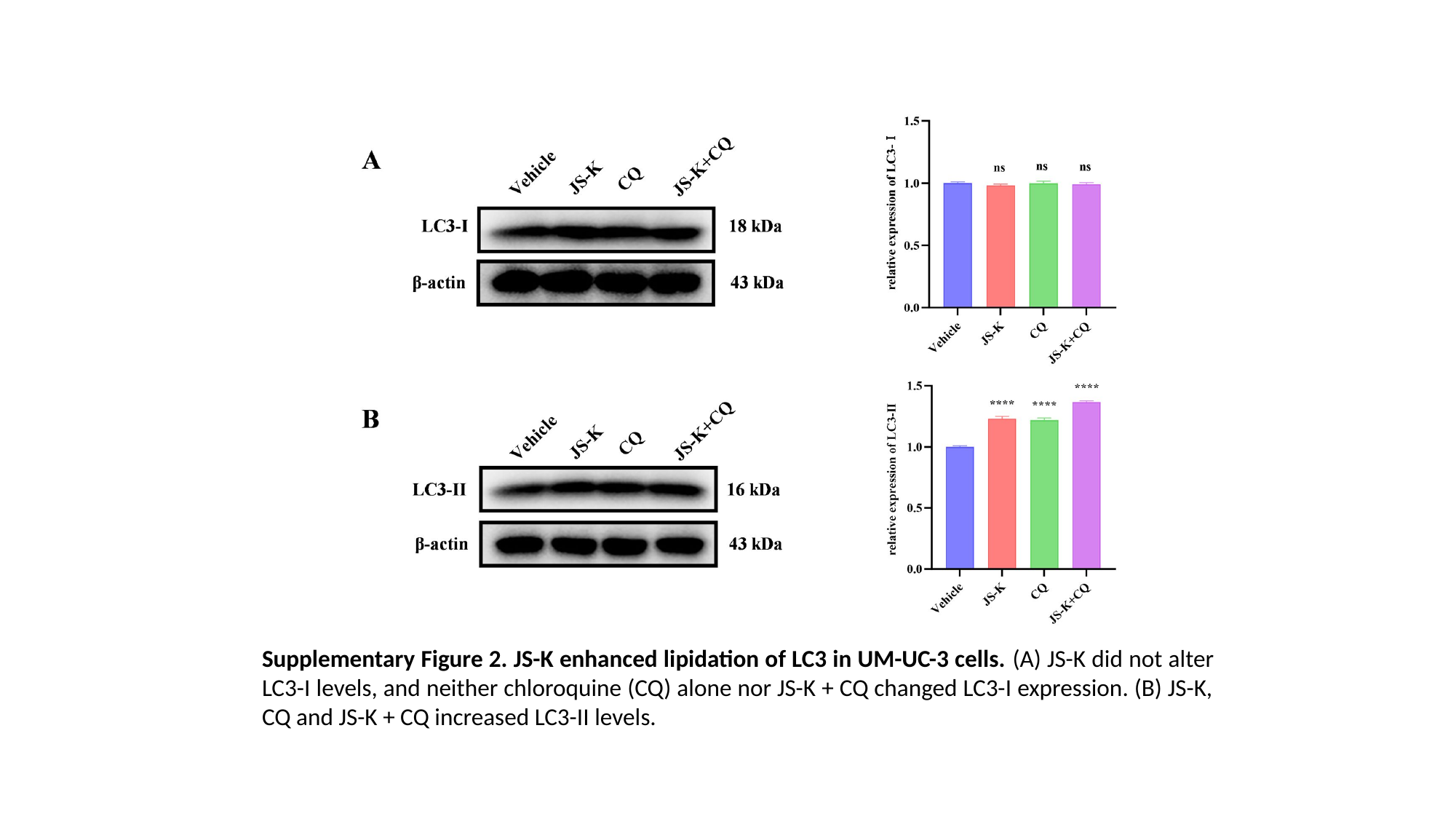

Supplementary Figure 2. JS-K enhanced lipidation of LC3 in UM-UC-3 cells. (A) JS-K did not alter LC3-I levels, and neither chloroquine (CQ) alone nor JS-K + CQ changed LC3-I expression. (B) JS-K, CQ and JS-K + CQ increased LC3-II levels.

## Slide 3
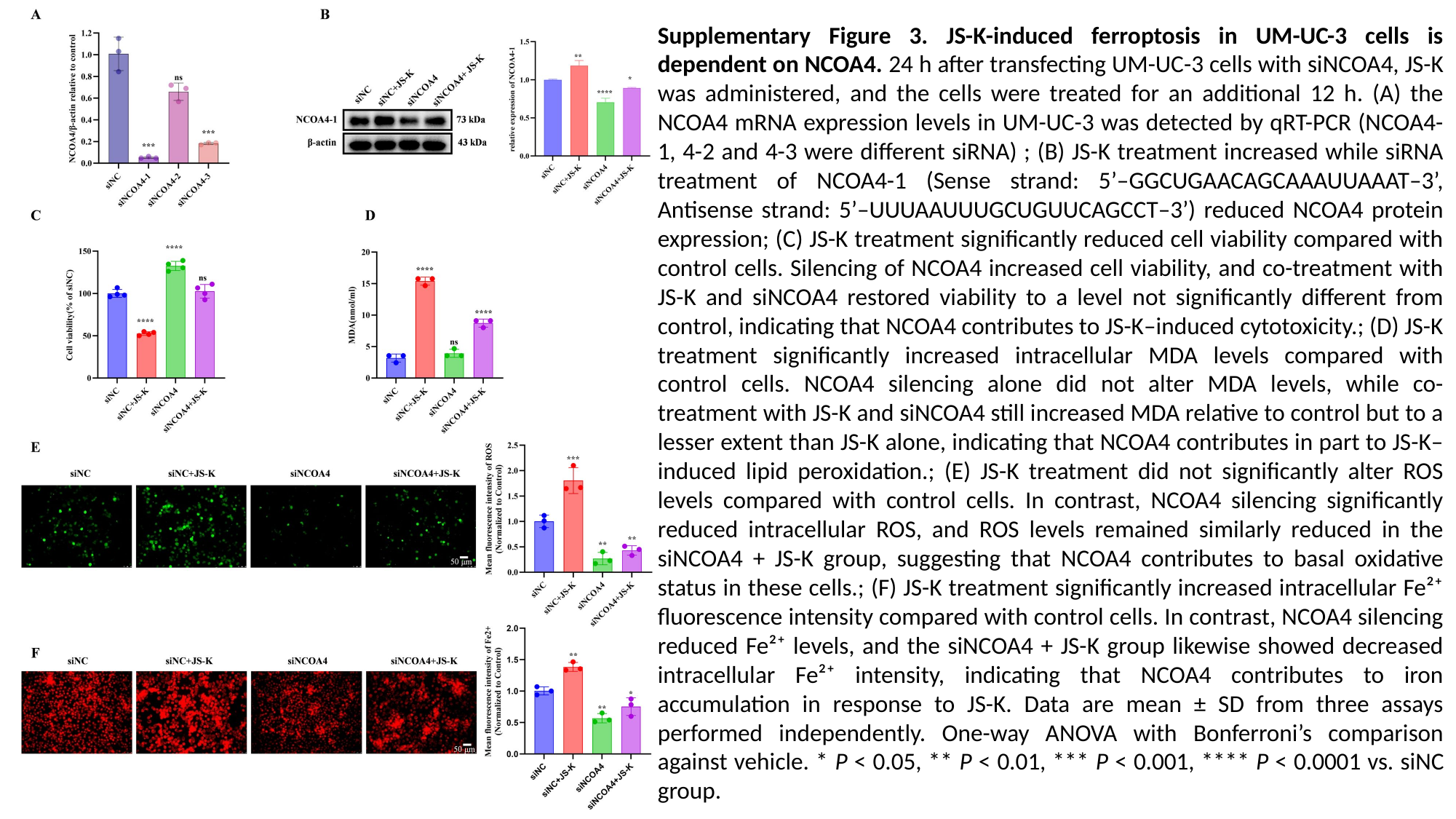

Supplementary Figure 3. JS-K-induced ferroptosis in UM-UC-3 cells is dependent on NCOA4. 24 h after transfecting UM-UC-3 cells with siNCOA4, JS-K was administered, and the cells were treated for an additional 12 h. (A) the NCOA4 mRNA expression levels in UM-UC-3 was detected by qRT-PCR (NCOA4-1, 4-2 and 4-3 were different siRNA) ; (B) JS-K treatment increased while siRNA treatment of NCOA4-1 (Sense strand: 5’–GGCUGAACAGCAAAUUAAAT–3’, Antisense strand: 5’–UUUAAUUUGCUGUUCAGCCT–3’) reduced NCOA4 protein expression; (C) JS-K treatment significantly reduced cell viability compared with control cells. Silencing of NCOA4 increased cell viability, and co-treatment with JS-K and siNCOA4 restored viability to a level not significantly different from control, indicating that NCOA4 contributes to JS-K–induced cytotoxicity.; (D) JS-K treatment significantly increased intracellular MDA levels compared with control cells. NCOA4 silencing alone did not alter MDA levels, while co-treatment with JS-K and siNCOA4 still increased MDA relative to control but to a lesser extent than JS-K alone, indicating that NCOA4 contributes in part to JS-K–induced lipid peroxidation.; (E) JS-K treatment did not significantly alter ROS levels compared with control cells. In contrast, NCOA4 silencing significantly reduced intracellular ROS, and ROS levels remained similarly reduced in the siNCOA4 + JS-K group, suggesting that NCOA4 contributes to basal oxidative status in these cells.; (F) JS-K treatment significantly increased intracellular Fe²⁺ fluorescence intensity compared with control cells. In contrast, NCOA4 silencing reduced Fe²⁺ levels, and the siNCOA4 + JS-K group likewise showed decreased intracellular Fe²⁺ intensity, indicating that NCOA4 contributes to iron accumulation in response to JS-K. Data are mean ± SD from three assays performed independently. One-way ANOVA with Bonferroni’s comparison against vehicle. * P < 0.05, ** P < 0.01, *** P < 0.001, **** P < 0.0001 vs. siNC group.

## Slide 4
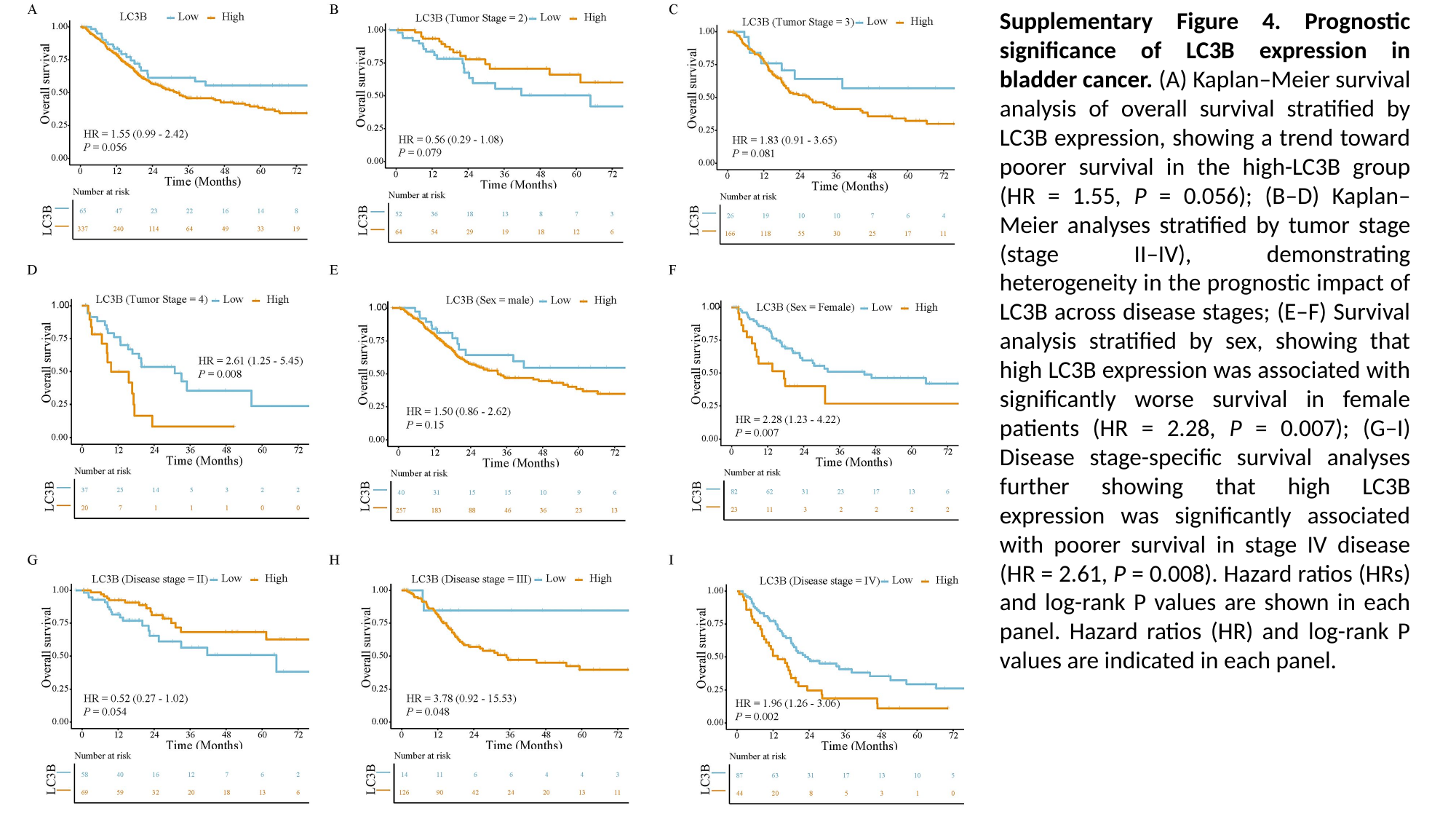

Supplementary Figure 4. Prognostic significance of LC3B expression in bladder cancer. (A) Kaplan–Meier survival analysis of overall survival stratified by LC3B expression, showing a trend toward poorer survival in the high-LC3B group (HR = 1.55, P = 0.056); (B–D) Kaplan–Meier analyses stratified by tumor stage (stage II–IV), demonstrating heterogeneity in the prognostic impact of LC3B across disease stages; (E–F) Survival analysis stratified by sex, showing that high LC3B expression was associated with significantly worse survival in female patients (HR = 2.28, P = 0.007); (G–I) Disease stage-specific survival analyses further showing that high LC3B expression was significantly associated with poorer survival in stage IV disease (HR = 2.61, P = 0.008). Hazard ratios (HRs) and log-rank P values are shown in each panel. Hazard ratios (HR) and log-rank P values are indicated in each panel.

## Slide 5
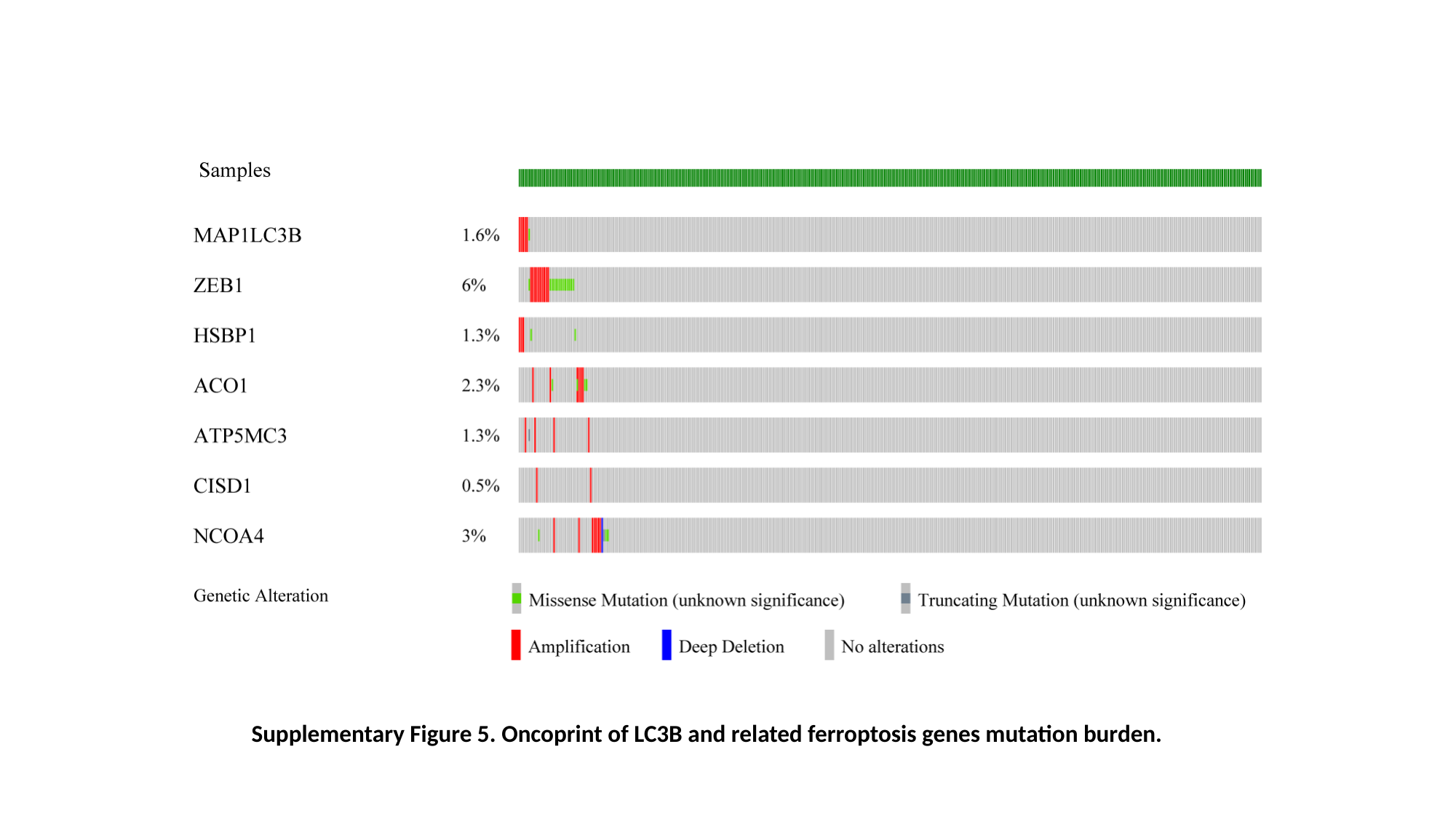

Supplementary Figure 5. Oncoprint of LC3B and related ferroptosis genes mutation burden.

## Slide 6
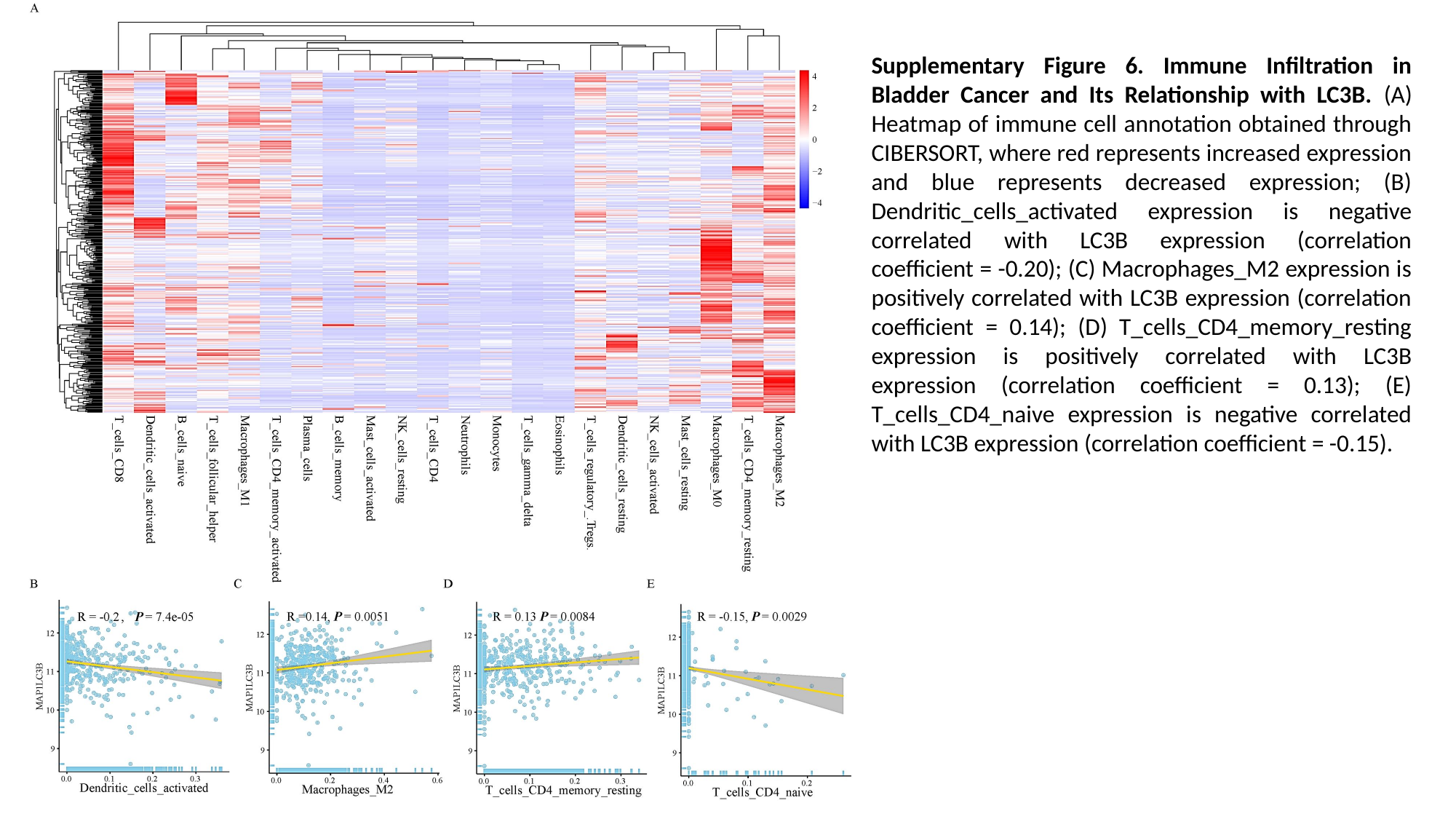

Supplementary Figure 6. Immune Infiltration in Bladder Cancer and Its Relationship with LC3B. (A) Heatmap of immune cell annotation obtained through CIBERSORT, where red represents increased expression and blue represents decreased expression; (B) Dendritic_cells_activated expression is negative correlated with LC3B expression (correlation coefficient = -0.20); (C) Macrophages_M2 expression is positively correlated with LC3B expression (correlation coefficient = 0.14); (D) T_cells_CD4_memory_resting expression is positively correlated with LC3B expression (correlation coefficient = 0.13); (E) T_cells_CD4_naive expression is negative correlated with LC3B expression (correlation coefficient = -0.15).
